# Prostate Cancer – Local Treatment after Radiorecurrence: HIFU – High-Intensity Focused Ultrasound

**DOI:** 10.1590/S1677-5538.IBJU.2018.03.03

**Published:** 2018

**Authors:** Stênio de Cássio Zequi, Thiago Camelo Mourão, Gustavo Cardoso Guimarães

**Affiliations:** 1 Divisão de Urologia, AC Camargo Cancer Center, Fundação A. Prudente, São Paulo, SP, Brasil; 2 Departamento de Urooncologia, Laparoscopia e Robótica, AC Camargo Cancer Center, Fundação A. Prudente, São Paulo, SP, Brasil

**Keywords:** High-Intensity Focused Ultrasound Ablation, Prostatic Neoplasms, Radiotherapy

Currently, about one third of all newly diagnosed prostate cancer patients select radiotherapy or brachytherapy (BT) as their primary treatment (1). Primary external beam radiation therapy (EBRT) in localized prostate cancer has a risk of biochemical recurrence about 30-60% (1, 2). The most widely utilized criteria for EBRT biochemical relapse is the Phoenix definition (2006). It is established as a PSA elevation of ≥ 2 ng/mL above the nadir PSA (3). There are a variety of treatment options, like watchful waiting, androgen deprivation therapy (ADT) and local salvage therapies. None of them are accepted as gold standard salvage treatments. Another important point is that recurrences are associated with an increased risk of death, metastases and local complications, such as ureteral obstruction, hematuria and pelvic pain (1).

Recent scenario shows that about 70% of these patients receive ADT, but with a decreasing trend over the time (4). ADT is not a curative treatment and it is associated with significant side effects, such as cardiovascular events, sexual dysfunction, humor disabilities, loss of bone mineral density and muscular atrophy. Indeed, proper salvage treatments are mandatory, especially in patients with good health status. We think that, among this population of men underwent upfront ADT, it is possible to select patients which can be submitted to local salvage procedures.

Salvage treatments for local recurrences are adequate after excluded systemic disease with a confirmatory biopsy sample of prostatic tissue and imaging modalities, such as magnetic resonance imaging, computed tomography, bone scan or even combined nuclear medicine techniques (choline PET/CT and PSMA PET/CT). Salvage local treatments are characterized by significant morbidity, with increased risk of rectal injuries and post-treatment incontinence or urethral stenosis that may be as high as 50% (5).

Salvage radical prostatectomy (SRP) is an accepted challenging alternative, due to the fact it is associated with a high morbidity rate, however less than 1% of patients in that situation receive this kind of approach (4). Reports have shown a 5-year and 10-year biochemical recurrence free rates of 47-82% and 28-53%, respectively, and a 5-year and 10-year cancer specific survival of 70-83% and 54-89%, respectively (6). A recent review by Golbari et al. evaluated the complication rates of SRP. Urinary incontinence varied from 20%-78%. Bladder neck contracture/urethral stenosis occurred in up to 42%. Rectal injury varied from 0-12.5% and erectile dysfunction, in the majority of studies analyzed, occurred in more than 70% and up 100% of the cases (1).

In 2011, Chade et al. reported a retrospective study with 404 radio-recurrent prostate cancer patients submitted to SRP. Pre-salvage PSA and post-RT prostate biopsy Gleason score were the main prognostic factors for biochemical failure and metastases (7).

The eligibility criteria demand an expert team, a long-life expectation, comorbidities and an initial favorable case (mobile prostate in digital rectal exam, no severe rectitis or cystitis). If patients present severe urinary dysfunctions secondary to chronic actinic complications, additional surgical procedures, such as urinary diversion, or bladder augmentation e.g. can be necessary.

The motivation to develop more feasible salvage local therapies has brought us the alternatives of salvage cryoablation and salvage brachytherapy. Reports about salvage cryotherapy have shown acceptable oncologic outcomes with a biochemical recurrence-free survival (BRFS) rate ranging from 28 to 87% (1). Additionally, these reports presented lower complication rates compared to SRP. Lian et al. analyzed the results of salvage cryotherapy with a third-generation technology after radiation therapy. The results were a 5-year BRFS of 43.5%, a urinary incontinence rate of 12% and erectile dysfunction in 57% (8). Another larger study reported urethral stricture in 5.5%, a bladder-outlet obstruction requiring transurethral resection (TURP) in about 4% and the necessity of sling or artificial urinary sphincter (AUS) placement due to urinary incontinence in almost 3%. Related to the performed technique, perineal pain is a possible problematic complication of cryotherapy occurring in up to 8% (9).

High-intensity focused ultrasound (HIFU) is a minimally invasive local ablative technique used either in the primary setting or in the salvage treatment (S-HIFU) of prostate cancer. First reports with this technique occurred in the 1990s. Currently, there are three commercially available devices (Sonablate – Focus Surgery Inc., Ablatherm – EDAP-TMS SA and Focal One – EDAP-TMS SA). It uses a transrectal piezoelectric ceramic transducer focusing on the target that causes thermal, mechanical and cavitation effects, producing a coagulative necrosis in the prostate (9). Temperatures achieved are above 80°C and there are safety features like a cooling system maintaining rectal mucosa at lower temperatures, a real time 7.5 Mhz. ultrasound visual supervision, continuous measurement of distance between rectal wall and the target, several security alarm levels, and a patient movement sensor (with millimetric sensibility).

S-HIFU is a minimally invasive procedure, requiring regional anesthesia in the vast majority of patients, with virtually no bleeding or associated post operatory thromboembolic events. The majority of the patients are discharged on the first operatory day. In this way, older or patients unfit to undergo a major procedure as SRP, could be candidates for S-HIFU prostatic ablation. Patients refractory to surgery can find in S HIFU an option for their recurrences after radiation therapy.

Factors implicating the prediction of progression are higher pre-EBRT PSA, higher pre-salvage HIFU PSA, PSA nadir > 0.5 ng/ mL after S-HIFU and shorter time until PSA nadir (1). Main contraindications for the procedure are inflammatory rectal disease, non-treated urinary tract infection, uncorrected coagulopathy, anal or rectal stenosis, rectal wall thickening > 8 mm (more common in irradiated patients), and prostate volume > 40 mL. Last situation, prostate must be down-sized by TURP before the procedure or even previous ADT (10).

After S-HIFU, European literature provides us some studies like Berge et al. that reported 46 patients showing a necessity of TURP for stricture/necrotic tissue in 4.4% and rectourethral fistula in just one case. Urinary incontinence grade II occurred in 15% and grade III, in 2% and erectile dysfunction was registered in 71% (10).

The largest series published to date on salvage HIFU therapy is by Crouzet et al. The group reported a mean 48-month follow-up with 290 patients submitted to whole-gland therapy. Median nadir PSA reported was 0.14 ng/mL and cancer-specific and metastasis-free survivals were 80% and 79%, respectively (11).

Siddiqui et al in Canada published a prospective trial showing a 5-year BDFS of 53% (in a series which only 37% of patients presented Gleason score ≤ 7 (3+4/ prognostic Group 2)), and the rest presented Gleason patterns 4, 5 (prognostic groups 3-5), or unknown) 3% (12). Previously, Murat reported BDFS of 73% in 18 months and Gelet et al. had a negative biopsy rate of 80% in 15 months using Ablatherm system (13, 14).

A recent North American prospective trial by Jones et al. with 100 patients reported that 50% achieved their 1-year endpoint of PSA nadir < 0.5 ng/mL and a negative biopsy (15). Dason et al. in another recent study reported no rectourethral fistulas, no osteitis pubis and just one case of urethral stricture on 24-eligible patients with a median follow-up of 31 months (6).

Last issues discuss the feasibility of the focal salvage HIFU which is based in the fact that about 66% of men who have localized failure after EBRT can develop recurrent unifocal or unilateral cancer, and the main site of recurrence is usually the site of the index lesion before EBRT (16). The recent advances of the prostatic multiparametric magnetic resonance and the image fusion techniques (with ultrasound) can help the planning of focal S-HIFU. In theory, focal treatments can result in less toxicity. Focal treatment can be either a quadrant ablation, a hemi-ablation or the index lesion ablation (with a surround security margin). Ahmed et al. in 2012 registered a BDFS of 49% in 2 years, an incontinence rate of 12.8% and a worsening of the International Index of Erectile Function-5 (IIEF-5) score from 18 ± 16 to 13 ± 21 at 6 months (16).

S-HIFU must be considered an important option in this setting. Selection of appropriate patients will provide a significant oncological control and lower rates of symptomatic complications compared to SRP. Patients with apical tumors should be informed of the risk of urinary incontinence (17). The best lower limit considered is about 6mm from the apex (10). Longer follow-up series and more prospective trials are important to support the current data.

At AC Camargo Cancer Center, between 2011-2018, from a total of 365 patients underwent HIFU, 37 (10.1%) were submitted to S-HIFU, being 35 (94.6%) whole gland S-HIFU and 2 (5.4%) focal S-HIFU; 29 (78%) cases corresponded to failures after EBRT and 8 (21%) after interstitial brachytherapy.

The mean age was 65 years old, the median pre operatory PSA, was 7.0 ng/mL. The median PSA nadir was 0.3 ng/mL. In 3-years of follow-up, 11 patients (29%) had recurrence. Biochemical and clinic failure were verified respectively in 9 (24.3%) and 2 patients (5.4%). There was not blood transfusion, or thromboembolic events. Main complications included urinary tract infections in 5 cases (13.5), urinary obstruction in 7 cases (18.9%) and rectal fistula in 1 case (2.7%).

In one patient procedure the procedure was aborted due to rectal wall thickening > 8 mm, and in other one, due to insurmountable anal stenosis.

In summary, there is an increase amount of patients presenting failure after EBRT or brachytherapy. In the future, with aging of population, this cluster of prostate cancer patients will become greater. Many of them are not adequate candidates to SRP, and many others are refractory to this challenging surgery and its inherent side effects. For these cases, S-HIFU (focal or whole gland ultrasonic ablation) is a safe, effective and minimally invasive procedure that can result in satisfactory biochemical control of the disease, or can postpone ADT and its adverse repercussions in male health.
